# Supplementary material for: Effects of Chronic Kidney Disease on Nanomechanics of the Endothelial Glycocalyx Are Mediated by the Mineralocorticoid Receptor
Source: Int J Mol Sci. 2022 Sep 13;23(18):10659. doi: 10.3390/ijms231810659 (PMC9503126; doi:10.3390/ijms231810659)
Supplement: Supplementary file 1 [file ijms-23-10659-s001.zip › ijms-1842619-supplementary.pdf]

Supplemental Table S1: Patient Characteristics (4C Study) Sera obtained at the baseline visit of 25 patients were used and patient characteristics are listed. The estimated glomerular filtration rate (mL/min/1.73 m2) of these patients was 41.3 in patients (n=10) with stage 3, 17.5 in patients (n=8) with stage 4, and 12.6 in patients (n=7) with stage 5 CKD (all pre-dialysis). Abbreviations: SDS= standard deviation score, eGFR= estimated glomerular filtration rate, cIMT= carotid intima-media thickness, PWV= pulse wave velocity, IS= indoxyl-sulfate, PCS= p-cresyl-sulfate, Angpt= Angiopoietin.^

	CKD G3<br>n= 10	CKD G4<br>n= 8	CKD G5<br>n= 7
Age (years)	13 ± 2.5	13 ± 3.6	13 ± 2.6
Height SDS	-0.1 ± 1.8	-1.6 ± 0.88	-1.3 ± 1.2
BMI SDS	0.081 ± 1.4	- 0.06 ± 1.5	-0.06 ± 0.87
eGFR (mL/min/1.73 m2)	41.3 ± 10	17.5 ± 1.8	12.6 ± 2.2
cIMT SDS	2.1 ± 2.0	2.1 ± 0.87	2.5 ± 2.7
PWV SDS	0.05 ± 1.7	0.16 ± 1.3	0.94 ± 1.4
Log IS (μmol/l)	0.87 ± 1.1	2.3 ± 1.3	2.7 ± 0.8
Log PCS (μmol/l)	2.3 ± 0.87	2.7 ± 0.91	3.4 ± 0.67
Angpt 1 (pg/mL)	39286 ± 10921	32749 ± 12500	49891 ± 25702
Angpt 2 (pg/mL)	3442 ± 1469	4956 ± 3307	6880 ± 5505
